# Supplementary material for: Microbial Pathway Thermodynamics: Stoichiometric Models Unveil Anabolic and Catabolic Processes
Source: Life (Basel). 2024 Feb 9;14(2):247. doi: 10.3390/life14020247 (PMC10890395; doi:10.3390/life14020247)
Supplement: Supplementary file 1 [file life-14-00247-s001.zip › life-2782096-supplementary.pdf]

Supplementary information - Microbial pathway thermodynamics: stoichiometric models unveil anabolic and catabolic processes

Oliver Ebenhöh Josha Ebeling Ronja Meyer Fabian Pohlkotte
Tim Nies

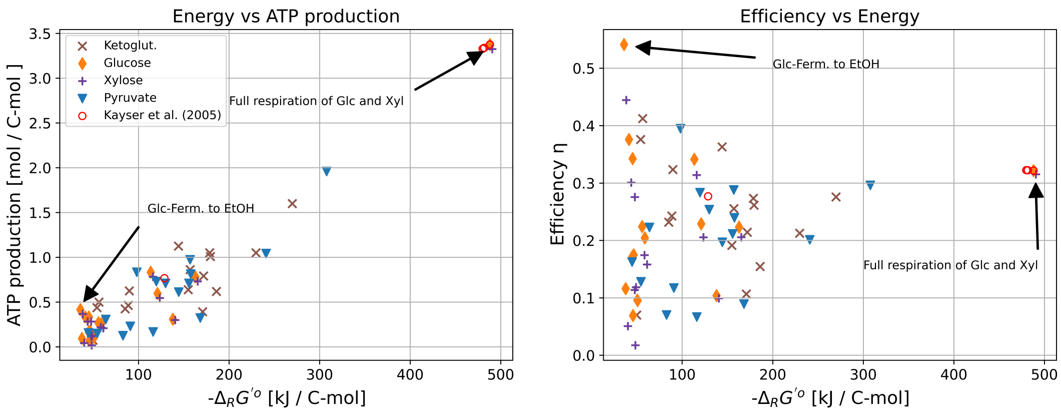

Figure S1: Thermodynamic characterisation of catabolic routes in *E. coli* genome-scale model (iJR904) for α-ketoglutarate, glucose, xylose, and pyruvate as carbon source. Additionally, oxygen is allowed to be a substrate in the calculation of the elementary conversion modes. The efficiency is based on a typical value of 46.5 kJ/mol for production of ATP in *E. coli* [43]. Only ECMs are plotted with a ATP yield higher than 0.001 mol/ C-mol.

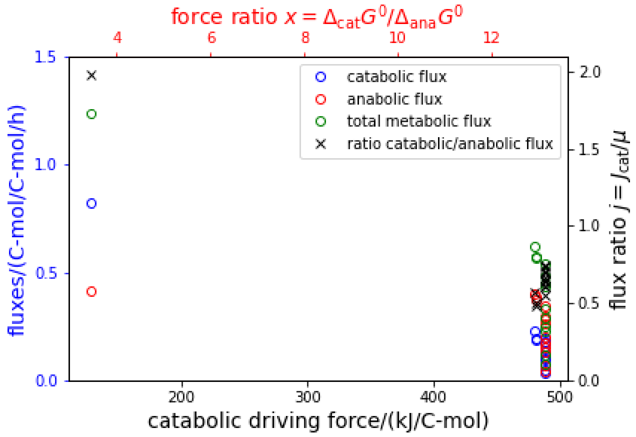

Figure S2: Metabolic fluxes as function of the catabolic driving force for data for *Escherichia coli* [37].Shown are the catabolic (blue), anabolic (red) and total (green) glucose consumption rates in dependence of the catabolic driving force, $-\Delta_{cat}G^0$. On the x-axis on the top, the force ratio $x = \Delta_{cat}G^0/\Delta_{ana}G^0$ is given.
